# Improved Production and Antitumor Properties of Triterpene Acids from Submerged Culture of *Ganoderma lingzhi*

**DOI:** 10.3390/molecules21101395

**Published:** 2016-10-20

**Authors:** Xiao-Ling Wang, Zhong-Yang Ding, Gao-Qiang Liu, Hailong Yang, Guo-Ying Zhou

**Affiliations:** 1 Key Laboratory of Cultivation and Protection for Non-Wood Forest Trees, Ministry of Education, Central South University of Forestry and Technology, Changsha 410004, China; cathy8311@163.com (X.-L.W.); gyzhouedu@aliyun.com (G.-Y.Z.); 2 Hunan Provincial Key Laboratory for Control of Forest Diseases and Pests, College of Life Science and Technology, Central South University of Forestry & Technology, Changsha 410004, China; 3 National Engineering Laboratory for Cereal Fermentation Technology, Jiangnan University, Wuxi 214122, China; qinxinhuzhu@tom.com; 4 College of Life and Environmental Science, Wenzhou University, Wenzhou 325035, China; yanzhaocsuft@sina.com

**Keywords:** antitumor activity, *Ganoderma lingzhi*, medicinal fungi, response surface methodology, submerged batch fermentation, triterpene acids

## Abstract

Triterpene acids (TAs) are the major bioactive constituents in the medicinal fungus *Ganoderma lingzhi*. However, fermentative production of TAs has not been optimized for commercial use, and whether the TAs isolated from *G. lingzhi* submerged culture mycelia possess antitumor activity needs to be further proven. In this study, enhanced TA yield and productivity were attained with *G. lingzhi* using response surface methodology. The interactions of three variables were studied using a Box-Benhnken design, namely initial pH, dissolved oxygen (DO) and fermentation temperature. The optimum conditions were an initial pH of 5.9, 20.0% DO and 28.6 °C. These conditions resulted in a TA yield of 308.1 mg/L in a 5-L stirred bioreactor. Furthermore, the optimized conditions were then successfully scaled up to a production scale of 200 L, and maximum TA production and productivity of 295.3 mg/L and 49.2 mg/L/day were achieved, which represented 80.9% and 111.5% increases, respectively, compared with the non-optimized conditions. Additionally, the triterpene acid extract (TAE) from *G. lingzhi* mycelia was found to be cytotoxic to the SMMC-7721 and SW620 cell lines in vitro, and the TAE exhibited dose-dependent antitumor activity against the solid tumor sarcoma 180 in vivo. Chemical analysis revealed that the key active triterpene compounds, ganoderic acid T and ganoderic acid Me, predominated in the extract.

## 1. Introduction

*Ganoderma* (Ganodermaceae) is a genus of edible and medicinal mushroom that is known as “reishi” in Japanese and “lingzhi” in Chinese [1]. The medicinal values of this mushroom were documented thousands of years ago [1]. Among the species of the genus, *G. lucidum* (Leyss. ex Fr.) Karst was originally described as coming from Europe and is now known in East Asia, Europe and North America. Recently, a new species *G. lingzhi* Sheng H. Wu, Y. Cao & Y.C. Dai, which is distributed in East Asia, was proposed. The most striking characteristics that differentiate *G. lingzhi* from *G. lucidum* are the presence of 1–2 black melanoid bands in the context, a yellow pore surface and thick dissepiments (80–120 μm) at maturity (in contrast, *G. lucidum* has a white pore surface and thin dissepiments (40–80 μm)) [2]. Modern chemistry has demonstrated that *Ganoderma* species contain a variety of phytochemicals. Two biologically active compounds that have been demonstrated to possess diverse and potentially significant pharmacological activities are polysaccharides and triterpenes (especially triterpene acids, TA) [1,3,4,5,6,7,8,9,10,11,12,13,14,15,16]. Recent studies have revealed that *Ganoderma* TA has a few important pharmacological activities that include antitumor effects, inhibition of cholesterol synthesis, anti-HIV-1 and anti-HIV-protease activities, and inhibition of U46619-induced platelet aggregation [5,6,7,8,9,10].

Currently, the submerged fermentation of *Ganoderma* is viewed as a promising approach for the efficient production of these polysaccharides and triterpenes because it is fast, cost-effective, and it is easy to control the product quality [11,12,13,14,15,16,17,18,19]. Many researchers have focused on the fermentation conditions and fermentation modes to stimulate the production of *Ganoderma* polysaccharides [11,12,13,14,15]. These polysaccharides have been used in traditional medicine as antitumor and immunomodulating agents [3,20]. In contrast, the triterpenes or TA products of *Ganoderma* are scarce on the international market, especially the pure individual triterpene compounds because the triterpene fermentation yield is very low and the required fermentation process is complex and not suitable for large-scale production [10,21]. Currently, few groups have studied triterpenes and specifically examined the production of ganoderic acids (a class of lanostane-type triterpenes) by *G. lucidum* [16,17,18,19], but data on the submerged fermentation of TA with other *Ganoderma* species are relatively scarce. Therefore, to meet the demand for TA for large-scale production, pharmacological testing and clinical applications, further study of TA production by *Ganoderma* is expected to improve the operability of the industrial application by a simple and feasible fermentation technology. Additionally, there are more than 100 kinds of triterpene compounds isolated from *Ganoderma* fruiting bodies, spores and mycelium (especially fruiting bodies). However, the constituents of the triterpenes from the three different sources are different, and most of the triterpenes or TAs with biological activities are isolated from the fruiting bodies of *Ganoderma* [4,22,23,24,25,26,27,28,29], and whether the triterpenes or TAs prepared from submerged culture *Ganoderma* (especially *G. lingzhi*) mycelium possesses antitumor activity needs to be further proved.

In statistics, response surface methodology (RSM) explores the relationships between several explanatory variables and one or more response variables, and the interactions between explanatory variables can be identified and quantified with this technique [30,31]. For a liquid rotation fermentation system for *Ganoderma*, the key fermentation conditions (explanatory variables or factors) for TA production (response variable) are the initial pH, fermentation temperature and dissolved oxygen (DO) [16,17,18,32]. Previous studies have mainly focused on an individual fermentation variable of pH or dissolved oxygen [16,17,18], and data about the relationships and interactions between several fermentation variables and TA production are scarce. Additionally, most studies have focused on *G. lucidum* [10,16,17,18,19,20], and data about the submerged fermentation of TAs with *G. lingzhi* are relatively limited. The aim of this work was to statistically optimize TA biosynthesis by the *Ganoderma* species *G. lingzhi*, which is distributed in East Asia, using RSM to develop a simple and feasible batch fermentation technology for large-scale TA production and to evaluate the in vitro and in vivo antitumor properties of the TA from submerged culture *G. lingzhi* mycelium.

## 2. Results

### 2.1. Characterization of the TA Extract from G. lingzhi Mycelia 

Based on the weight of the crude triterpene acid extract (TAE), the yield was approximately 6.1%. The total TA content of the extract was 21.0%. Previous studies have demonstrated that ganoderic acids A, T, Me, DM and lucidenic acid F isolated from the fruiting bodies or spores of *G. lucidum* exhibit antitumor activities [5,27,28,29,33,34], and ganoderic acid B exhibits inhibitory activity against HIV-1 protease [7]. Therefore, we determined the contents of these active TA compounds in the extract from the mycelium of *G. lingzhi*, and the results are displayed in Table 1.

The ganoderic acid T content was the highest (70.1 mg/g), followed by ganoderic acid Me (43.3 mg/g) and ganoderic acid A (8.2 mg/g). The extract also contains small amounts of ganoderic acid B, DM and lucidenic acid F. 

### 2.2. TA Production under Non-Optimized Fermentation Conditions

According to the fermentation media and the basic fermentation conditions, the *G. lingzhi* fermentation experiment was performed twice in a 5 L fermenter to verify the TA production yield. The results revealed that the biomass (mycelial dry weight, DW) reached 12.1 g/L, and a TA production yield of 163.3 mg/L and a per day productivity of 23.3 mg/L were obtained.

### 2.3. Optimization of TA Production by CENTRAL Composite Design and RSM

The key fermentation conditions for a liquid fermentation system of *G. lingzhi* are the initial pH, fermentation temperature and DO [16,17,18,32]. In the present work, the levels of the three key factors were further optimized with a Box-Benhnken design and RSM to improve the TA production yield. The ranges and the levels of the variables in the design are given in Table 2, and the results of the experiments are presented in Table 3.

#### 2.3.1. Analysis of Variance (ANOVA) and Model Fitting

To evaluate the relationships between the TA response and the independent fermentation variables and to determine the maximum TA production that corresponds to the optimal initial pH (*x*_1_), DO (*x*_2_, %), and temperature (*x*_3_, °C), a second-order polynomial model was proposed. By applying multiple regression analysis to the experimental data, a second-order polynomial model in coded units explained the role of each variable and their second-order interactions on TA production. The quadratic model of the response equation in terms of the coded variables was as follows:

*Y*_TA_ = 283.58 + 43.52*x*_1_ + 3.74*x*_2_ + 6.39*x*_3_ − 2.48*x*_1_*x*_2_ − 0.28*x*_1_*x*_3_ − 7.72*x*_2_*x*_3_ − 26.06*x*_1_^2^ − 14.73*x*_2_^2^ − 10.78*x*_3_^2^ (1)

where *Y*_TA_ is the predicted TA yield, *x*_1_ is the initial pH, *x*_2_ is the DO, and *x*_3_ is the temperature.

ANOVA of the quadratic regression model revealed that the model was highly significant, as evidenced by the low *p* value for the Fisher’s *F*-test ((*p*_model_ > *F*) < 0.0001) (Table 4). This finding proved that the model of Equation (1) is a suitable model for describing the experimental TA production response. The closer *R*^2^ is to 1.00, the stronger the model is and the better it predicts the response [31]. In this case, the coefficient of determination was *R*^2^ = 0.9910, which implied that TA production was attributable to the independent variables. This *R*^2^ also indicated that less than 1% of the total variation was not explained by the model. These measures indicate that the accuracy and general ability of the polynomial model were good and that the analysis of the response trends using the model was reasonable.

The ANOVA results for all factors are presented in Table 5. The linear term *x*_1_ (initial pH) had an extremely significant effect on TA production (*p* < 0.01), followed by *x*_3_ (temperature) (*p* < 0.05). 

The term *x*_2_ (DO) exhibited no marked effect on TA production. The quadratic terms of all three variables indicated extremely negative quadratic effects on mycelial TA yield (*p* < 0.01). The interactive term of *x*_2_*x*_3_ was extremely significant at *p* < 0.01, which suggests that DO and temperature had a direct relationship with TA production.

#### 2.3.2. Interactions between the Factors and Selection of Optimum Levels

**Figure 1:**
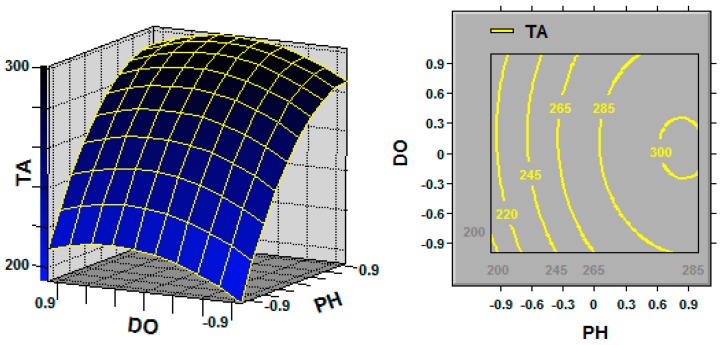
Response surface curve (**left**) and contour plot (**right**) for triterpene acid (TA; mg/L) production illustrating the interaction between the initial pH (PH) and dissolved oxygen (DO) at the coded levels. Fixed level: temperature = 0.

**Figure 2:**
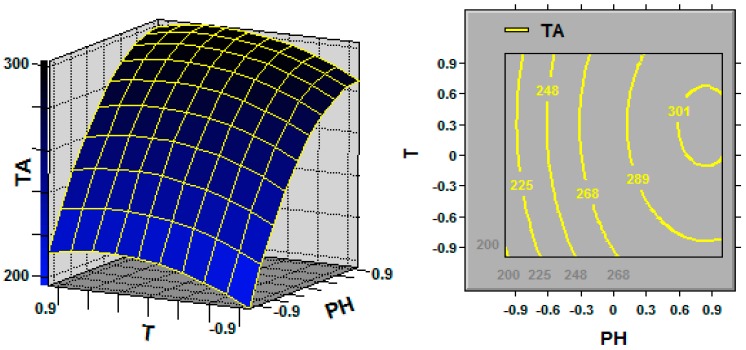
Response surface curve (**left**) and contour plot (**right**) for triterpene acid (TA; mg/L) production illustrating the interaction between the initial pH (PH) and temperature (T) at the coded levels. Fixed level: DO = 0.

**Figure 3:**
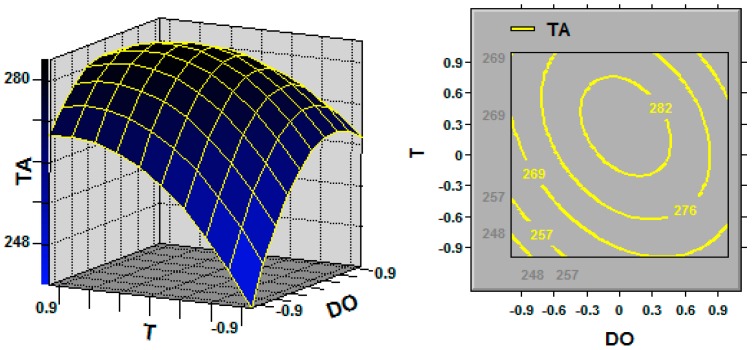
Response surface curve (**left**) and contour plot (**right**) for triterpene acid (TA; mg/L) production illustrating the interaction between the dissolved oxygen (DO) and temperature (T) at the coded levels. Fixed level: initial pH = 0.

The 3D response surface and the 2D contour plots described by the regression model were drawn to illustrate the effects of the independent variables and interactive effects of each independent variable on the response variables. Figure 1, Figure 2 and Figure 3 represent the 3D response surfaces and their corresponding contour plots for the optimization of TA production. Each figure presents the effect of two variables on TA production while another variable was held at the zero level (coded value).

From the response surface plots, it is easy to understand the interactions between two factors. As illustrated in Figure 1, increasing the coded DO level to 0.3 led to a slight increase in TA production; however, upon increasing the initial pH to 0.8, the TA production significantly increased. Another pair of the independent variables (i.e., initial pH and temperature; Figure 2) exhibited effects similar to those apparent in Figure 1.

Figure 3 illustrates the combined effects of dissolved oxygen (DO) and temperature (T) on TA production. TA production was obviously sensitive to even small alterations in DO and temperature, and the TA yield was high when the DO level was in the intermediate range and the coded temperature level was in the range of 0 to 0.3.

TA production evidently reached its maximum at the combination of coded levels of 0.834 (x_1_, initial pH), −0.020 (x_2_, DO) and 0.292 (x_3_, temperature) based on canonical analysis with SAS software. The model predicted a maximum response of 302.6 mg TA/L at the optimized fermentation conditions of an initial pH of 5.9, DO 20.0% and temperature 28.6 °C.

#### 2.3.3. Verification of the Model

**Figure 4:**
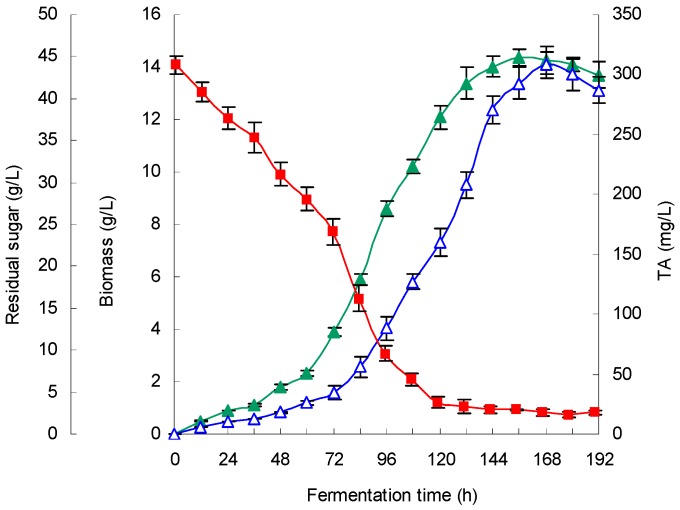
Time courses of sugar consumption (■), mycelial growth (▲) and mycelial triterpene acid (TA) production (△) during *Ganoderma lingzhi* growth in a 5 L stirred-tank bioreactor under the optimized fermentation conditions. Medium volume, 3.5 L; initial pH, 5.9; fermentation temperature, 28.6 °C; DO, ~20.0%; aeration rate, 0.3 vvm; culture time, 8 days.

Triplicate experiments in a 5 L fermenter culture were performed to verify the availability and accuracy of the model (Equation (1)). Mycelial growth time profiles, TA production and substrate consumption are presented in Figure 4. 

Under the optimized fermentation conditions, cell growth exhibited a similar exponential phase from 60 h to 156 h, and the biomass (dry cell weight) reached a maximum concentration of 14.4 g/L at 156 h. Residual sugar concentration decreased sharply to 3.8 g/L at 120 h and to 2.6 g/L at 168 h. The TA synthesis lagged behind cell growth, and a maximum TA yield of 308.1 mg/L was attained at 168 h, which represented an 88.7% increase in the titer compared with that of the non-optimized conditions (163.3 mg/L). This finding was also in agreement with the predicted value (302.6 mg/L), which suggests that the model (Equation (1)) was very valid for TA production. 

#### 2.3.4. Verification of the Model Using a Large-Scale Fermenter 

**Figure 5:**
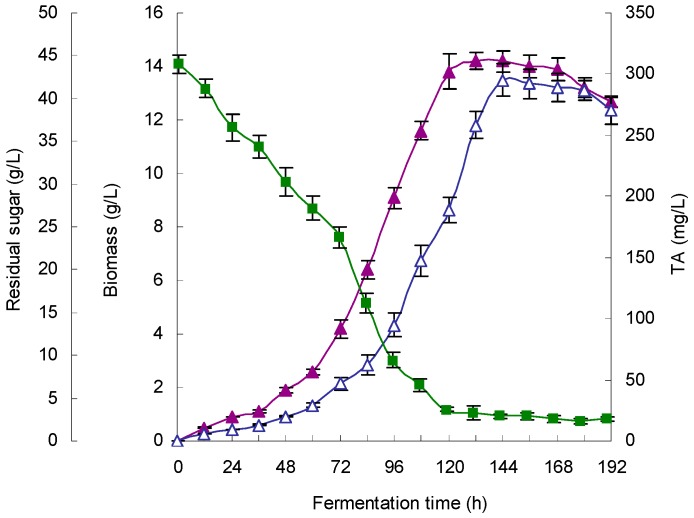
Time profiles of sugar consumption (■), mycelial growth (▲) and mycelial triterpene acid (TA) production (△) by *Ganoderma lingzhi* under the optimized fermentation conditions in a 200-L stirred-tank bioreactor. Medium volume, 140 L; initial pH, 5.9; DO, ~20%; temperature, 28.6 °C; aeration rate, 0.7 vvm; culture time, 8 days.

The feasibility of the regression model was further tested in a 200-L scale fermenter under the optimized fermentation conditions. Figure 5 shows the time courses of mycelial growth, TA production and substrate consumption. This figure demonstrates that the concentrations of residual sugar in the 200-L scaled bioreactor sharply decreased to 3.7 g/L at 120 h and 2.9 g/L at 156 h. Biomass and TA production increased earlier in the 200-L fermenter, and the TA concentrations were maximal at 144 h (6th day) with a yield of 295.3 mg/L, which represented an 80.9% increase compared to that in the non-optimized conditions (163.3 mg/L). This result was also close to the predicted value (302.6 mg/L) in the 5-L fermenter culture, which suggests that the model (Equation (1)) was also valid for TA production on a large scale. Moreover, the productivity of TA in the 200-L scaled fermenter was significantly enhanced to 49.2 mg/L per day, which represented a 111.5% increase compared to that of the non-optimized conditions (23.3 mg/L per day).

### 2.4. In Vitro Antitumor Activity of TAE

**Figure 6:**
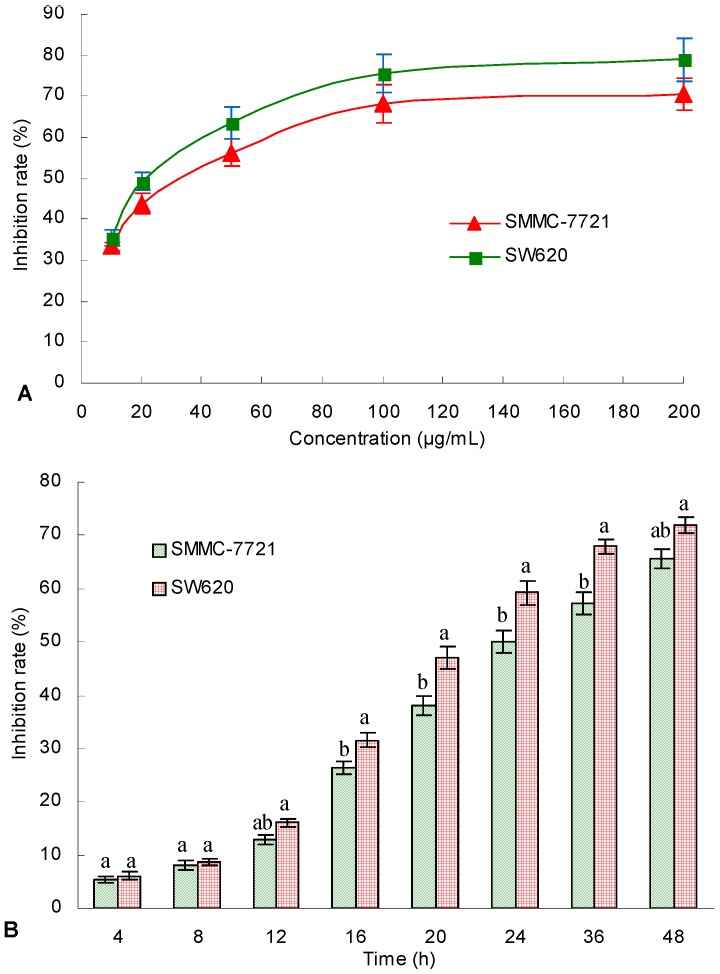
In vitro antitumor activities of triterpene acid extract (TAE) from the mycelium of *Ganoderma lingzhi* against human hepatoma SMMC-7721 and human colon carcinoma SW620 cells at different concentrations (**A**) or at different treatment times (**B**) with a final concentration of 33.1 μg/mL TA. The data points are the means ± standard deviations (*n* = 3). Different letters indicate significant (*p* < 0.05) differences between treatments.

The in vitro cytotoxicities of the produced triterpene acid extract (TAE) to human hepatoma SMMC-7721 and human colon carcinoma SW620 cells were analyzed (Figure 6). When the TA concentrations increased, the inhibition rates against both the SMMC-7721 and SW620 cells significantly increased (Figure 6A). TA at 100 μg/mL induced inhibition rates of 68.3% and 75.6% against the SMMC-7721 and SW620 cells, respectively, after 24 h of incubation. The IC_50_ values for TAE against the SMMC-7721 and SW620 cells were 33.1 and 21.3 μg/mL, respectively, after 24 h of treatment.

The inhibitory effects of the TAE treatment times at the concentration of 33.1 μg/mL on the growths of the SMMC-7721 and SW620 cells are illustrated in Figure 6B. With increasing treatment time, the inhibition rates against the SMMC-7721 and SW620 cells significantly increased. TAE exhibited the same inhibitory effects against the two cell lines in the first 8 h (*p* > 0.05) and induced greater mortality against the SW620 cells than the SMMC-7721 cells when the treatment time reached 12 h or longer (*p* < 0.05). These results indicate that the crude TAE was cytotoxic to human hepatoma SMMC-7721 and human colon carcinoma SW620 cells, and the SW620 cells were more sensitive to the TA from submerged culture *G. lingzhi* mycelium.

### 2.5. In Vivo Antitumor Activity of TAE

**Figure 7:**
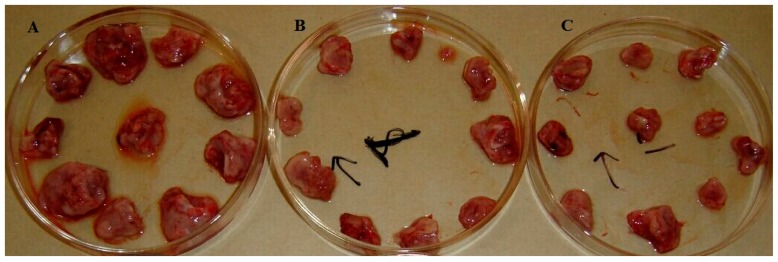
Morphological features of the sarcoma 180 solid tumors that were removed from the Kunming mice in the control (**A**), triterpene acid extract (TAE, 250 mg/kg) (**B**) and 5-FU (**C**) treatment groups (10 mice per group).

To determine whether TA has an antitumor effect in vivo, a sarcoma 180 solid tumor model was established by subcutaneously injecting S-180 cells into Kunming mice. Table 6 illustrates that obvious and dose-dependent antitumor activities were observed at all doses (62.5, 125 and 250 mg/kg). Furthermore, TAE (125 and 250 mg/kg) obviously enhances the thymus index and spleen index values. Figure 7 visually illustrates the morphological features of the sarcoma 180 solid tumors that were removed from the mice in the control, TAE (250 mg/kg) and 5-FU treatment groups. The tumor sizes in the TAE (250 mg/kg) treatment group were markedly smaller than those in the control group. 

## 3. Discussion

Dissolved oxygen (DO) has an important influence on the aerobic microbial fermentation process of. Oxygen supply affects cell growth, nutrient uptake and metabolite biosynthesis. Typically, sufficient oxygen supply results in an increase in the specific growth rate; however, the relationship between oxygen supply and metabolite biosynthesis is not clear [33,34,35,36]. In the cultivation of the filamentous fungus *Schizophyllum commune*, a sufficient oxygen supply results in a decrease in the production of extracellular glucan [33], whereas in the submerged fermentation of *Monascus ruber*, improving the oxygen supply increases both the biomass and secondary metabolite (red pigment and citrinin) yields [34]. Regarding *Ganoderma* fermentation, one study reported that the production of ganoderic acids (a class of lanostane-type triterpenes) and polysaccharides by *G. lucidum* are significantly enhanced in 2-L stirred-tank bioreactors when the DO is maintained between 20% and 35% air saturation [35]. Another study revealed that the production of the extracellular polysaccharides of *G. lucidum* under constant DO control at 10% is higher than that under 25% DO [36]. In the present study, we first examined the effects of DO on TA production by *G. lingzhi*; the TA yield was increased by maintaining the DO at 20% over the entire fermentation process, and the cell growth was also promoted by maintaining the DO at 20%. 

Environmental factors, such as culture pH and temperature, can greatly affect metabolite production. Both of these factors can affect the uptake of various nutrients and the activity of important enzymes associated with metabolite biosynthesis [37,38,39,40], and the culture pH can also affect the cell membrane function [37,38,39]. Regarding *Ganoderma* fermentation, Fang and Zhong [37] found that at an initial pH of 6.5, a maximum biomass of 17.3 g/L as well as a maximal production of ganoderic acid (a class of lanostane-type triterpenes) of 207.9 mg/L were obtained in a shake-flask culture of *G. lucidum* at 30 °C, and the suitable initial pH and temperature for *G. lingzhi* fermentation were found to be 5.9 and 28.6 °C, respectively, in this work, and a TA production of 308.1 mg/L was obtained in a 5-L stirred bioreactor.

The scaling up of key fermentation factors is essential for potential commercial applications. There are some reports about large-scale submerged mushroom fermentation [41,42]. The scaling up of the filamentous fungus *Glarea lozoyensis* from a pilot scale (0.07 m^3^, 0.8 m^3^, and 19 m^3^) to a production scale (57 m^3^) to produce the pharmaceutically important compound pneumocandin has been successfully completed by maintaining a constant DO [41]. In the submerged fermentation of *Streptomyces gilvosporeus* LK-196, the dissolved oxygen level and culture pH are also considered to be two key factors in the scaling up from 30- and 1000- to 18,000-L, and a high level of natamycin production was reproduced with the control of the dissolved oxygen level over 30% and a constant pH of 7.0 [42]. Tang et al. developed a good fermentation strategy involving a pH shift and a DOT shift integrated into a fed-batch fermentation process for total ganoderic acid production by *G. lucidum* [17], and this process was successfully step-wise scaled-up from a 7.5- to a 200-L stirred-tank bioreactor [43]. Nevertheless, previous studies on *Ganoderma* have mainly focused on an individual fermentation factor, i.e., pH or dissolved oxygen [16,17,18,35,36,37], and the interactions between the fermentation variables were not been investigated. In the present work, the relationships between several fermentation variables (i.e., pH, dissolved oxygen and temperature) and TA production and the interactions between the fermentation variables were analyzed by RSM. The TA was examined following a simple batch fermentation process, and it was found to efficiently accumulate in the *G. lingzhi* mycelium following statistical optimization of the key fermentation parameters. Moreover, the optimized process conditions were successfully reproduced in 5-L stirred-tank bioreactors and were also scaled up to a 200-L fermenter while the DO was controlled at 20.0%, the pH at 5.9 and the temperature at 28.6 °C. Furthermore, the *G. lingzhi* fermentation time (6 days) in the 200-L scaled bioreactor was much shorter than that (18 days) of the fed-batch fermentation process for *G. lucidum* that has been reported in the literature [17]. Thus, the batch fermentation process was simpler, which may make the process easier to apply in large-scale production.

*Ganoderma* triterpenes have been reported to be cytotoxic to several cancer cell lines in vitro and to inhibit tumor invasion in vitro and in vivo [4,5,6,27,28,29,44,45,46,47]. Currently, most of the triterpenes and TAs with reported antitumor activities are isolated from the fruiting bodies or spores of *Ganoderma* (mainly *G. lucidum*) [4,6,27,28,29,47]. Regardless of whether the triterpenes or TAs are prepared from submerged culture *Ganoderma* (especially *G. lingzhi*) mycelium, the potential antitumor activities of these compounds need to be further proven. Recently, a few works regarding triterpenes (or ganoderic acids) prepared from the mycelium of *G. lucidum* have been completed. One study demonstrated a triterpene-enriched fraction, WEES-G6, prepared from the mycelium of *G. lucidum* inhibits the growth of human hepatoma Huh-7 cells by suppressing protein kinase C, activating mitogen-activated protein kinases and arresting the cell cycle in the G2 phase [48]. Another work indicated that ganoderic acid T isolated from *G. lucidum* mycelium inhibits the proliferation of human colon carcinoma HCT-116 cells [5]. Furthermore, three new ganoderic acid T derivatives display marked inhibitory effects on the growth of human cervical cancer HeLa cells, cause cell cycle arrest at the G1 phase and induce apoptosis [49]. Ganoderic acid Me isolated from *G. lucidum* mycelium inhibits proliferation, angiogenesis, and invasion and induces apoptosis in MDA-MB-231 cells [50], and it also induces the apoptosis of competent T cells and increases the proportion of Treg cells [51]. In this work, TA from the mycelium of *G. lingzhi* was studied. For the first time, the TAE from submerged culture *G. lingzhi* mycelium was found to be cytotoxic to the human hepatoma SMMC-7721 and human colon carcinoma SW620 cell lines. Furthermore, the TAE (125 mg/kg and 250 mg/kg) from *G. lingzhi* mycelium obviously enhances thymus index and spleen index values in vivo, which suggests that the TA from *G. lingzhi* mycelium might enhance body cell immune function at a certain dose. Hence, the antitumor effect of TA from *G. lingzhi* mycelium is probably host-mediated and cytocidal. Additionally, previous studies have demonstrated that triterpene compounds, including ganoderic acids Me, T, DM and lucidenic acid F from *G. lucidum*, have strong antitumor activities [5,28,29,44,45]. Our results suggest that the TAE from the mycelium of *G. lingzhi* also contains the typical TA compounds isolated from the fruiting bodies of *Ganoderma* and that ganoderic acids T and Me dominantly exist in the extract. Therefore, the TAE from *G. lingzhi* may have antitumor activities that are related to its high ganoderic acid T and Me contents. The identification of the other active principles and the elucidation of their mechanisms of action remain to be achieved. 

In conclusion, enhanced TA production yield and productivity were attained with *G. lingzhi* via the optimization of the batch fermentation conditions. The TAE from *G. lingzhi* mycelium has strong active principle contents and possesses antitumor activity in vitro and in vivo.

## 4. Materials and Methods

### 4.1. Chemicals and Media

Penicillin, streptomycin, dimethylsulfoxide (DMSO), RPMI 1640 and fetal bovine serum were obtained from Gibco BRL Co. (Gaithersburg, MD, USA). 5-Fluorouracil (5-FU) was obtained from Sigma Chemical Co. (St. Louis, MO, USA). Ganoderic acids A, B, DM, lucidenic acid F and ursolic acid were purchased from Nanjing Puyi Biotech Co. (Nanjing, China). Ganoderic acid T and Me were extracted and purified from mycelium with preparative liquid chromatography in our lab to purities over 96%. All other chemicals were analytical-grade reagents.

### 4.2. Seed Culture

*G. lingzhi* SCIM 1005 was kindly provided by Prof. Yu-Cheng Dai of the Institute of Microbiology, Beijing Forestry University, Beijing, China, and stored in the Strain Collection of Industrial Microorganisms (SCIM), Central South University of Forestry & Technology (Changsha, China). The strain was isolated from the fruiting body specimen, which is the sample of the identified new species *G. lingzhi* in the published datum (reference [2]). The original fruiting body specimen is deposited at Beijing Forestry University, Beijing, China. The strain was cultured in a 500-mL flask containing 170 mL seed culture medium (see below) at 27 °C for 8 days with shaking at 160 r/min [10]. The seed culture medium was composed of the following (in g/L): glucose (44.0), corn flour (0.5), peptone (6.5), KH_2_PO_4_ (0.75), MgSO_4_·7H_2_O (0.45) and vitamin B_1_ (0.01).

### 4.3. Culture in the 5-L Bioreactor

The seed was inoculated at 10% (*v*/*v*) into a 5-L bioreactor (New Brunswick Scientific Co., Inc., Edison, NJ, USA) under different initial pH, DO and temperature conditions for the optimization of TA production. 

The basic fermentation conditions were as follows: medium volume 3.5 L, inoculation volume 10% (*v*/*v*), initial pH 5.5, fermentation temperature 27 °C, DO ~ 15%, and culture time 7 days. The DO level (percentage of air saturation) was controlled at a constant value (~10%, ~20%, ~30%) by cascading different agitation speeds (from 150 up to 500 rpm), and the ventilation was 0.3 vvm throughout. The reactor fermentation was repeated twice, and the data shown represent the mean values with the standard deviations.

### 4.4. Fermentation Process in the 200-L Agitated Fermenter

Large-scale fermentation was performed with a method similar to that described in our previous work [31] in an agitated 200-L fermenter (ABEC, Inc., Bethlehem, PA, USA) under the following conditions: medium volume 140 L, inoculation volume 10% (*v*/*v*), initial pH 5.9, fermentation temperature 28.6 °C, DO ~20.0%, and culture time 8 days. The DO level (percentage of air saturation) was controlled at a constant value by cascading different agitation speeds (from 150 up to 500 rpm), and the ventilation was 0.7 vvm throughout. The fermentation was repeated twice, and the values are expressed as the mean ± standard deviation.

### 4.5. Determination of the Dry Cell Weight

The determination of the dry cell biomass was performed by a method described in our previous work [31].

### 4.6. Measurement of TA

The determination of total TA was performed according to a method described in a previous work with slight modifications [16]. The dried mycelia (2 g) were extracted by circumfluence with 75% (*v*/*v*) ethanol (100 mL) for 3 h (twice). After removal of the mycelia by centrifugation, the supernatant was dried at 50 °C under vacuum. The residues were suspended in water and later extracted with chloroform (100 mL) for 2 h (twice). After removal of the chloroform by evaporation at 40 °C, the sample was further extracted by 5% (*w*/*v*) NaHCO_3_ (200 mL) for 12 h. After adding 2 M HCl to adjust the pH of the NaHCO_3_ layer to 3, the triterpenes in the NaHCO_3_ layer were extracted with chloroform (200 mL) for 12 h. After removal of the chloroform by evaporation at 40 °C, the TA was then dissolved in absolute ethanol, and its absorbance was measured at 245 nm using ursolic acid as the standard [16].

### 4.7. Preparation of the Triterpene Acid Extract (TAE)

The mycelium of *G. lingzhi* was dried at 60 °C and ground to a fine powder. The powder samples were refluxed in five volumes of 75% (*v*/*v*) ethanol for 3 h (twice). After removal of the mycelium by centrifugation, the supernatant was dried at 50 °C under a vacuum. The residues were suspended in water and later extracted with five volumes of chloroform for 2 h (twice). After removal of the chloroform by evaporation at 40 °C, the TA was further extracted with five volumes of 5% (*w*/*v*) NaHCO_3_ for 12 h (twice). After adding 2 M HCl to adjust the pH of the NaHCO_3_ layer to 3.0, the TA in the NaHCO_3_ layer was extracted with five volumes of chloroform for 12 h (twice). The chloroform layer was evaporated at 40 °C under reduced pressure to yield the TAE. 

### 4.8. Chemical Properties of the TAE

The yield of crude TA was measured as a percentage of the total weight of sample used. The determination of the individual triterpene compounds (ganoderic acid A, B, T, Me, DM and lucidenic acid F) was performed according to the HPLC method as described in a previous work [52]. 

### 4.9. RSM Experimental Design and Statistical Analysis

Considering that the TA yield was low under the basic fermentation conditions (i.e., the non-optimized conditions), a Box-Benhnken design was used for optimization of the design of the TA fermentation to enhance the TA yield. According to previous works [16,17,18,32], the appropriate pH, DO and temperature for TA production are in the approximate ranges of 5.0–6.0, 10%–30% air saturation and 26–30 °C, respectively. Therefore, the lowest and highest levels of the variables in the Box-Benhnken design were set as follows: initial pH, 5.0 and 6.0; DO, 10% and 20%; and temperature, 26 °C and 30 °C (Table 1). For all variables, the central coded value was considered as zero. The Box-Benhnken design matrix and the TA responses are illustrated in Table 2. A mathematical model describing the relationships between the TA yields and the fermentation parameters in a second-order equation was developed. The TA yield from *G. lingzhi* was subjected to multiple regression with respect to the fermentation parameters by the least squares method as in Equation (2):

*Y*_i_ = *β*_0_ + ∑ *β*_i_*x*_i_ + ∑ *β*_ii_*x*_i_*^2^* + ∑ *β*_ij_*x*_i_*x*_j_ (2)

where *Y*_i_ is the predicted response variable; *β*_0_, *β*_i_, *β*_ii_, and *β*_ij_ are the constant regression coefficients of the model; and *x*_i_ and *x*_j_ (*i* = 1, 3; *j* = 1, 3, *i* ≠ *j*) represent the independent variables (fermentation parameters) in the form of the coded values.

The Statistical Analysis System (SAS v.8.1, Cary, NC, USA) was used for the analysis of variance, regression and graphical analysis. The accuracy and general ability of the above polynomial model was evaluated with the coefficient of determination *R*^2^ [31]. The significance of all factors was determined using t-tests. The optimum levels for the initial pH, temperature and DO were obtained by solving the regression equation [31].

### 4.10. In Vitro Studies

#### 4.10.1. Tumor Cells

Human hepatoma SMMC-7721 and human colon carcinoma SW620 cell lines were purchased from the Shanghai Institute of Cell Biology (Shanghai, China). The cells were cultured in RPMI-1640 medium supplemented with 15% fetal bovine serum, 100 U/mL penicillin and 100 U/mL streptomycin at 37 °C in a 5% CO_2_ atmosphere. The cells were seeded in 96-well plates (Costar) at an initial density of (5–7) × 10^5^ cells/mL and incubated for 24 h [53]. 

#### 4.10.2. In Vitro Antitumor Activity Assay for TAE

An in vitro antitumor activity assay was performed according to the method of Xiao et al. with slight modifications [53]. The cytotoxicity of TAE was detected with a 3-[4,5-dimethylthiazol-2-yl]-2,5-diphenyl tetrazolium bromide (MTT) assay using human hepatoma SMMC-7721 and human colon cancer SW620 cells. The cells were seeded into 96-well plates (0.8 × 10^4^ cells per well) 24 h prior to the assay, and then the cells were treated for 24 h with a series of doses of TAE. Non-treated cells were used as controls. After incubation, the medium was removed, and fresh medium (160 μL) was added to each well. Subsequently, 40 μL of 5 mg/mL MTT solution was added to each well, and the plate was incubated for 4 h. The medium was then carefully discarded, and acidified ethanol (100 μL) was added to each well. The optical density (OD) was then measured at 570 nm with a DG-3022A ELISA reader (Nanjing, China). The cytotoxicity of triterpene acid was expressed as the relative cell mortality (inhibition rate) calculated as [1 − (OD_sample_/OD_control_) × 100%]. The experiments were conducted in triplicate, and the values are expressed as the means ± the standard deviations. The Statistical Analysis System software (SAS v.8.1, Cary, NC, USA) was used for the analysis of variance, and significant differences were estimated by Duncan’s multiple range tests at a probability level of 0.05. 

### 4.11. In Vivo Studies 

#### 4.11.1. Animals

Four-week-old Kunming male mice (18–22 g) were purchased from Nanjing Qinglongshan Experimental Animal Breeding Center, Nanjing, China. The animals were fed a commercial diet (containing 19.2% protein, 5.2% fat, 57.1% carbohydrates, 3.7% fiber, 6.2% minerals, and 8.7% water) in a room at 25 ± 2 °C. Food and water were available ad libitum. 

#### 4.11.2. Experimental Design and Antitumor Activity Assay

The in vivo antitumor activity assay was performed according to a method described in the literature [54]. Sarcoma 180 solid tumors were initially supplied and maintained by Jiangsu Tumor Hospital, Nanjing, China. The tumor cells (3 × 10^7^–5 × 10^7^ cells/mL) were injected subcutaneously (s.c.) into the right armpit region of the Kunming male mice at 0.2 mL per mouse. The animals were randomized into a control group and 5-FU and TAE treatment groups (10 per group). The TAE solid dispersions were prepared with poloxamer-188 (F68, BASF, Ludwigshafen, Germany), and the TAE samples were orally administered to the tumor-bearing mice at 62.5 mg/kg, 125 mg/kg and 250 mg/kg once daily for 10 days beginning 24 h after tumor inoculation. The same volume of saline and 5-FU (25 mg/kg once daily) was orally administered to the mice in the control and 5-FU treatment groups, respectively. On day 10, the mice were sacrificed, and the tumors, spleen and thymus were harvested and weighed. The inhibition ratio, spleen index and thymus index values were calculated according to a method described in the literature [54]. The Statistical Analysis System (SAS v.8.1, Cary, NC, USA) was used for analysis of variance, and significant differences were estimated with Duncan’s multiple range tests.

## Figures and Tables

**Table 1:** Triterpene acid compound contents of the triterpene acid extract (TAE) from *G. lingzhi* mycelium.

Triterpene Acid Compounds	Contents (mg/g)
Ganoderic acid A	8.2 ± 0.4
Ganoderic acid B	5.7 ± 0.3
Ganoderic acid DM	0.2 ± 0.0
Ganoderic acid T	70.1 ± 4.7
Ganoderic acid Me	43.3 ± 2.7
Lucidenic acid F	0.1 ± 0.0

The values are presented as the mean ± SD, *n* = 3.

**Table 2:** Experimental ranges and levels of the independent variables for mycelial TA optimization.

Runs	Coded Values			*Y*_TA_ (mg/L)
	Factor x_1_	Factor x_2_	Factor x_3_	
1	−1	−1	0	192.4
2	−1	1	0	208.2
3	1	−1	0	282.3
4	1	1	0	288.3
5	0	−1	−1	240.8
6	0	−1	1	271.3
7	0	1	−1	260.3
8	0	1	1	260.0
9	−1	0	−1	196.7
10	1	0	−1	286.3
11	−1	0	1	207.7
12	1	0	1	296.2
13	0	0	0	277.1
14	0	0	0	281.9
15	0	0	0	290.7
16	0	0	0	279.2
17	0	0	0	289.1

**Table 3:** The Box-Benhnken design matrix and the mycelial TA responses of *G. lingzhi*.

Independent Variables	Range and Levels		
	−1	0	1
Initial pH, x_1_	5.0	5.5	6.0
DO, x_2_ (%)	10.0	20.0	30.0
Temperature, x_3_ (°C)	26.0	28.0	30.0

**Table 4:** Analysis of variance (ANOVA) for the full quadratic model for optimization of mycelial TA production in *G. lingzhi*.

Regression	DF	Type I Sum of Squares	R-Square	*F* value	*Pr* > F
Linear	3	15590	0.7526	195.2000	<0.0001 **
Quadratic	3	4674.5419	0.2257	58.5300	<0.0001 **
Cross product	3	263.3698	0.0127	3.3000	0.0877
Total model	9	20527	0.9910	85.6800	<0.0001 **

** Significant at the 0.01 level.

**Table 5:** Analysis of variance (ANOVA) for all factors for optimization of TA production by *G. lingzhi*.

Parameter	DF	Estimate	Standard Error	*t* Value	*Pr* > |t|
Intercept	1	283.5800	2.3074	122.90	<0.0001 **
*x*_1_	1	43.5188	1.8242	23.86	<0.0001 **
*x*_2_	1	3.7388	1.8242	2.05	0.0796
*x*_3_	1	6.3900	1.8242	3.50	0.0100 *
x_1_^2^	1	−26.0600	2.5145	−10.36	<0.0001 **
*x*_1_*x*_2_	1	−2.4750	2.5798	−0.96	0.3693
x_2_^2^	1	−14.7250	2.5145	−5.86	0.0006 **
*x*_1_*x*_3_	1	−0.2825	2.5798	−0.11	0.9159
*x*_2_*x*_3_	1	−7.7225	2.5798	−2.99	0.0201 *
x_3_^2^	1	−10.7825	2.5145	−4.29	0.0036 **

* Significant at the 0.05 level, ** Significant at the 0.01 level.

**Table 6:** Antitumor activity of triterpene acid extract (TAE) from *Ganoderma lingzhi* against sarcoma 180 solid tumors in mice and the immune effects of TAE.

Measurements	Group				
	Control	5-FU	TAE		
Dose (mg/kg days)	-	25	62.5	125	250
Tumor weight (g)	1.6 ± 0.7	0.7 ± 0.4 **	1.2 ± 0.3 *	1.0 ± 0.2 **	0.9 ± 0.2 **
Inhibition ratio (%)	-	54.0 ± 3.1	24.2 ± 2.7	37.3 ± 6.2	46.6 ± 4.6
Thymus index (mg/g)	2.5 ± 1.3	2.5 ± 0.9	2.6 ± 0.4	2. 9 ± 0.7 *	3.1 ± 0.5 *
Spleen index (mg/g)	5. 9 ± 1.2	7.9 ± 1.7 *	6.0 ± 1.4	7.0 ± 0.6 *	7.6 ± 1.3 **

The values are the mean ± SD, *n* = 10; * Significantly different from the control by Duncan test (*p* < 0.05), ** *p* < 0.01.

## Abbreviations

The following abbreviations are used in this manuscript:

*G. lucidum*	*Ganoderma lucidum*
*G. lingzhi*	*Ganoderma lingzhi*
RSM	Response surface methodology
T	temperature
TA (TAs)	Triterpene acids
TAE	Triterpene acid extract

## References

[1] Liu G.Q., Zhang K.C. (2015). Mechanisms of the anticancer action of *Ganoderma lucidum* (Leyss. ex Fr.) Karst.: A new understanding. J. Integr. Plant. Biol..

[2] Cao Y., Wu S.H., Dai Y.C. (2012). Species clarification of the prize medicinal *Ganoderma* mushroom “Lingzhi”. Fungal Divers..

[3] Habijanic J., Berovic M., Boh B., Plankl M., Wraber B. (2015). Submerged cultivation of *Ganoderma lucidum* and the effects of its polysaccharides on the production of human cytokines TNF-α, IL-12, IFN-γ, IL-2, IL-4, IL-10 and IL-17. New Biotechnol..

[4] Gao J.J., Min B.S., Ahn E.M., Nakamura N., Lee H.K., Hattori M. (2002). New triterpene aldehydes, lucialdehydes A-C, from *Ganoderma lucidum* and their cytotoxicity against murine and human tumor cells. Chem. Pharm. Bull..

[5] Chen N.H., Liu J.W., Zhong J.J. (2010). Ganoderic acid T inhibits tumor invasion in vitro and in vivo through inhibition of MMP expression. Pharmacol. Rep..

[6] Min B.S., Gao J.J., Nakamura N., Hattori M. (2010). Triterpene acids from the spores of *Ganoderma lucidum* and their cytotoxicity against meth-A and LLC tumor cells. Chem. Pharm. Bull..

[7] El-Mekkawy S., Meselhy M.R., Nakamura N., Tezuka Y., Hattori M., Kakiuchi N., Shimotohno K., Kawahata T., Otake T. (1998). Anti-HIV-1 and anti-HIV-1-protease substances from *Ganoderma lucidum*. Phytochemistry.

[8] Miyamoto I., Liu J., Shimizu K., Sato M., Kukita A., Kukita T., Kondo R. (2009). Regulation of osteoclastogenesis by ganoderic acid DM isolated from *Ganoderma lucidum*. Eur. J. Pharmacol..

[9] Chen Y.S., Ming S.S., Cheng T.W. (1999). Differential effects of ganodermic acid S on the thromboxane A2-signaling pathways in human platelets. Biochem. Pharmacol..

[10] Wang XL., Yang H., Liu GQ. (2016). Enhanced triterpene acid production by *Ganoderma lucidum* using a feeding stimulus integrated with a two-stage pH-control strategy. J. Chem. Technol. Biot..

[11] Liu G.Q., Zhang K.C. (2007). Enhancement of polysaccharides production in *Ganoderma lucidum* by the addition of ethyl acetate extracts from *Eupolyphaga sinensis* and *Catharsius molossus*. Appl. Microbiol. Biotechnol..

[12] Ji S.L., Liu R., Ren M.F., Li H.J., Xu J.W. (2015). Enhanced production of polysaccharide through the overexpression of homologous uridine diphosphate glucose pyrophosphorylase gene in a submerged culture of lingzhi or reishi medicinal mushroom, *Ganoderma lucidum* (higher basidiomycetes). Int. J. Med. Mushrooms.

[13] Xu J.W., Ji S.L., Li H.J., Zhou J.S., Duan Y.Q., Dang L.Z., Mo M.H. (2015). Increased polysaccharide production and biosynthetic gene expressions in a submerged culture of *Ganoderma lucidum* by the overexpression of the homologous α-phosphoglucomutase gene. Bioprocess. Biosyst. Eng..

[14] Zhou H., Liu G., Huang F., Wu X., Yang H. (2014). Improved production, purification and bioactivity of a polysaccharide from submerged cultured *Ganoderma lucidum*. Arch. Pharm. Res..

[15] Hsieh C.Y., Hsu T.H., Yang F.C. (2005). Production of polysaccharides of *Ganoderma lucidum* (CCRC36021) by reusing thin stillage. Process. Biochem..

[16] Fang Q.H., Zhong J.J. (2002). Submerged fermentation of higher fungus *Ganoderma lucidum* for production of valuable bioactive metabolites—Ganoderic acid and polysaccharide. Biochem. Eng. J..

[17] Tang Y.J., Zhang W., Zhong J.J. (2009). Performance analyses of a pH-shift and DOT-shift integrated fed-batch fermentation process for the production of ganoderic acid and Ganoderma polysaccharides by medicinal mushroom *Ganoderma lucidum*. Bioresource. Technol..

[18] Zhang W.X., Zhong J.J. (2010). Effect of oxygen concentration in gas phase on sporulation and individual ganoderic acids accumulation in liquid static culture of *Ganoderma lucidum*. J. Biosci. Bioeng..

[19] Zhu L.W., Zhong J.J., Tang Y.J. (2010). Multi-fed batch culture integrated with three-stage light irradiation and multiple additions of copper ions for the hyperproduction of ganoderic acid and *Ganoderma* polysaccharides by the medicinal mushroom *Ganoderma lucidum*. Process. Biochem..

[20] Gao Y., Zhou S., Jiang W., Huang M., Dai X. (2003). Effects of ganopoly (a *Ganoderma lucidum* polysaccharides extract) on the immune functions in advanced-stage cancer patients. Immunol. Invest..

[21] Xu J.W., Zhao W., Zhong J.J. (2010). Biotechnological production and application of ganoderic acids. Appl. Microbiol. Biotechnol..

[22] Liu D.Z., Zhu Y.Q., Li X.F., Shan W.G., Gao P.F. (2014). New triterpenoids from the fruiting bodies of *Ganoderma lucidum* and their bioactivities. Chem. Biodivers..

[23] Lee I., Kim H., Youn U., Kim J., Min B., Jung H., Na M., Hattori M., Bae K. (2010). Effect of lanostane triterpenes from the fruiting bodies of *Ganoderma lucidum* on adipocyte differentiation in 3T3-L1 cells. Planta Med..

[24] Fatmawati S., Shimizu K., Kondo R. (2010). Ganoderic acid Df, a new triterpenoid with aldose reductase inhibitory activity from the fruiting body of *Ganoderma lucidum*. Fitoterapia.

[25] Lee I., Ahn B., Choi J., Hattori M., Min B., Bae K. (2011). Selective cholinesterase inhibition by lanostane triterpenes from fruiting bodies of *Ganoderma lucidum*. Bioorg. Med. Chem. Lett..

[26] Fatmawati S., Shimizu K., Kondo R. (2011). Ganoderol B: A potent α-glucosidase inhibitor isolated from the fruiting body of *Ganoderma lucidum*. Phytomedicine.

[27] Yao X., Li G., Xu H., Lü C. (2012). Inhibition of the JAK-STAT3 signaling pathway by ganoderic acid A enhances chemosensitivity of HepG2 cells to cisplatin. Planta. Med..

[28] Wu G.S., Lu J.J., Guo J.J., Li Y.B., Tan W., Dang Y.Y., Zhong Z.F., Xu Z.T., Chen X.P., Wang Y.T. (2012). Ganoderic acid DM, a natural triterpenoid induces DNA damage G1 cell cycle arrest and apoptosis in human breast cancer cells. Fitoterapia.

[29] Watanabe K., Shuto T., Sato M., Onuki K., Mizunoe S., Suzuki S., Sato T., Koga T., Suico MA., Kai H., Ikeda T. (2011). Lucidenic acids-rich extract from antlered form of *Ganoderma lucidum* enhances TNFα induction in THP-1 monocytic cells possibly via its modulation of MAP kinases p38 and JNK. Biochem. Biophys. Res. Commun..

[30] He S., Wang H., Wu B., Zhou H., Zhu P., Yang R., Yan X. (2013). Response surface methodology optimization of fermentation conditions for rapid and efficient accumulation of macrolactin A by marine *Bacillus amyloliquefaciens* ESB-2. Molecules.

[31] Liu G.Q., Wang X.L. (2007). Optimization of critical medium components using response surface methodology for biomass and extracellular polysaccharide production by *Agaricus blazei*. Appl. Microbiol. Biotechnol..

[32] Liu G.Q., Xiao H.X., Wang X.L., Zhao Y., Zhang Y.G., Ren G.P. (2011). Stimulated production of triterpenoids of *Ganoderma lucidum* by an ether extract from the medicinal insect, *Catharsius molossus* and identification of the key stimulating active components. Appl. Biochem. Biotechnol..

[33] Rau U., Gura E., Olszewski E., Wangner F. (1992). Enhanced glucan formation of filamentous fungi by effective mixing. Ind. Microbiol..

[34] Hajjaj H., Blane P.J., Groussac E., Goma G., Uribelarrea J.L., Loubiere P. (1999). Improvement of red pigment/citrinin production ratio as a function of environmental conditions by *Monascus ruber*. Biotechnol. Bioeng..

[35] Tang Y.J., Zhong J.J. (2002). Fed-batch fermentation of *Ganoderma lucidum* for hyperproduction of polysaccharide and ganoderic acid. Enzym. Microb. Technol..

[36] Tang Y.J., Zhong J.J. (2003). Role of oxygen supply in submerged fermentation of *Ganoderma lucidum* for production of Ganoderma polysaccharide and ganoderic acid. Enzym. Microb. Technol..

[37] Fang Q.H., Zhong J.J. (2002). Effect of initial pH on production of ganoderic acid and polysaccharide by submerged fermentation of *Ganoderma lucidum*. Process. Biochem..

[38] Hu Z.C., Zheng Y.G., Wang Z., Shen Y.C. (2006). pH control strategy in astaxanthin fermentation bioprocess by *Xanthophyllomyces dendrorhous*. Enzym. Microb. Technol..

[39] Liu S.R., Wu Q.P., Zhang J.M., Mo S.P. (2015). Efficient production of ε-poly-l-lysine by *Streptomyces ahygroscopicus* using one-stage pH control fed-batch fermentation coupled with nutrient feeding. J. Microbiol. Biotechnol..

[40] Feng J., Feng N., Zhang J., Yang Y., Wen J., Lin C.-C. (2016). A new temperature control shifting strategy for enhanced triterpene production by *Ganoderma lucidum* G0119 based on submerged liquid fermentation. Appl. Biochem. Biotechnol..

[41] Pollard D.J., Kirschner T.F., Hunt G.R., Tong I.-T., Stieber R., Salmon P.M. (2007). Scale up of a viscous fungal fermentation: Application of scale-up criteria with regime analysis and operating boundary conditions. Biotechnol. Bioeng..

[42] Liang J., Xu Z., Liu T., Lin J., Cen P. (2008). Effects of cultivation conditions on the production of natamycin with *Streptomyces gilvosporeus* LK-196. Enzym. Microb. Technol..

[43] Tang Y.J., Zhang W., Liu R.-S., Zhu L.-W., Zhong J.J. (2011). Scale-up study on the fed-batch fermentation of *Ganoderma lucidum* for the hyperproduction of ganoderic acid and *Ganoderma* polysaccharides. Process. Biochem..

[44] Chen N.H., Liu J.W., Zhong J.J. (2008). Ganoderic acid Me inhibits tumor invasion through down-regulating matrix metalloproteinases 2/9 gene expression. J. Pharmacol. Sci..

[45] Chen N.H., Zhong J.J. (2009). Ganoderic acid Me induces G1 arrest in wild-type p53 human tumor cells while G1/S transition arrest in p53-null cells. Process. Biochem..

[46] Amen YM., Zhu Q., Tran HB., Afifi MS., Halim AF., Ashour A., Mira A., Shimizu K.J. (2016). Lucidumol C, a new cytotoxic lanostanoid triterpene from *Ganoderma lingzhi* against human cancer cells. J. Nat. Med..

[47] Wu T.S., Shi L.S., Kuo S.C. (2001). Cytotoxicity of *Ganoderma lucidum* triterpenes. J. Nat. Prod..

[48] Lin S.B., Li C.H., Lee S.S., Kan L.S. (2003). Triterpene-enriched extracts from *Ganoderma lucidum* inhibit growth of hepatoma cells via suppressing protein kinase C, activating mitogen-activated protein kinases and G2-phase cell cycle arrest. Life Sci..

[49] Liu RM., Li YB., Zhong JJ. (2012). Cytotoxic and pro-apoptotic effects of novel ganoderic acid derivatives on human cervical cancer cells in vitro. Eur. J. Pharmacol..

[50] Li F., Wang Y., Wang X., Li J., Cui H., Niu M. (2012). Ganoderic acids suppress growth and angiogenesis by modulating the NF-κB signaling pathway in breast cancer cells. Int. J. Clin. Pharmacol. Ther..

[51] Que Z., Zou F., Zhang A., Zheng Y., Bi L., Zhong J., Tian J., Liu J. (2014). Ganoderic acid Me induces the apoptosis of competent T cells and increases the proportion of Treg cells through enhancing the expression and activation of indoleamine 23-dioxygenase in mouse lewis lung cancer cells. Int. Immunopharmacol..

[52] Zhou J.S., Ji S.L., Ren M.F., He Y.L., Jing X.R., Xu J.W. (2014). Enhanced accumulation of individual ganoderic acids in a submerged culture of *Ganoderma lucidum* by the overexpression of squalene synthase gene. Biochem. Eng. J..

[53] Xiao H., Yang T., Lin Q., Liu G.Q., Zhang L., Yu F., Chen Y. (2016). Acetylated starch nanocrystals: preparation and antitumor drug delivery study. Int. J. Biol. Macromol..

[54] Zhang L., Liu W., Han B., Sun J., Wang D. (2008). Isolation and characterization of antitumor polysaccharides from the marine mollusk *Ruditapes philippinarum*. Eur. Food Res. Technol..

